# Weight stigma among adolescents in three low- and middle-income countries

**DOI:** 10.7189/jogh.12.04098

**Published:** 2022-12-16

**Authors:** Ishu Kataria, Angela Jackson-Morris, Jo Jewell, D’Arcy Williams, Prince Bhandari, Deepika Sharma, Joanna Lai, Tanvi Jain, David Colozza

**Affiliations:** 1 Center for Global Noncommunicable Diseases, RTI International, North Carolina, USA; 2 UNICEF, New York, USA; 3 RTI International, New Delhi, India; 4 UNICEF, Jakarta, Indonesia

## Abstract

**Background:**

Overweight (OW) and obesity affect millions of adolescents worldwide. Evidence from high-income countries indicates widespread weight stigma that adversely affects young people’s mental and physical health. However, evidence relating to low- and middle-income countries (LMICs) is sparse. We aimed to generate insight into weight stigma prevalence and experience among adolescents in three LMICs.

**Methods:**

We identified adolescents aged 15-19 from Brazil, South Africa, and Indonesia from families within market research databases. We adopted a mixed-methods design. The sample included equal numbers by country, sex, and age, and included urban and rural dwellers. Self-reported weight was recorded but was not a selection criterion. Consent (age >18) and assent/parental consent (<age 18) were obtained. In-depth interviews (n = 18) informed the survey design. We used a computer-assisted telephonic interviewing survey (n = 1200) to assess weight stigma prevalence and experience. We conducted a qualitative thematic data analysis and used SPSS-23 for quantitative data.

**Results:**

Many adolescents affected by OW and some affected by underweight (UW) had experienced weight stigma and expressed negative perceptions of their weight. Negative perceptions were expressed the most by those affected by OW, followed by those affected by UW, and then those of healthy weight (HW). Weight stigma and weight dissatisfaction were highest in Brazil and South Africa, and lower in Indonesia. More young women experienced weight stigma, yet this was also common among young men. One in five of all participants reported negative impacts, predominantly on mental health, and regarding weight management and healthy behaviours. Young people recommended measures to address weight stigma, promote an inclusive model of health and body image, and better support to achieve and maintain a HW.

**Conclusions:**

Weight stigma adversely affects sizeable numbers of adolescents in LMICs, particularly those affected by OW. Addressing this is essential to protect adolescent (and future adult) mental and physical health.

Overweight (OW) and obesity among children and adolescents are a growing concern, affecting 340 million persons aged 5-19 worldwide in 2016 [1]. Once considered a high-income country (HIC) issue, the global epidemiological and nutrition transition over recent decades has produced a sharp escalation in prevalence in low-and middle-income countries (LMICs) [2]. Key shifts include an increased promotion, affordability, and consumption of energy-dense, nutrient-poor foods, reduced physical activity, and more sedentary behaviour.

High obesity prevalence in HICs has been accompanied by an increase in weight stigma [3]. According to Link and Phelan, “stigma is a powerful social process that is characterized by labeling, stereotyping, and separation, leading to status loss and discrimination, all occurring in the context of power” [4]. Research indicates that many children and adolescents in HICs experience weight stigma [5], irrespective of socioeconomic status, body size, race, or academic attainment [6,7]. Weight stigma can adversely affect adolescents’ physical and mental health (increasing risk of anxiety and depression and setting down cardiovascular risk factors) [8], may contribute to low educational attainment [9], and can undermine healthy weight (HW) management via binge eating, reduced physical activity, and increased weight gain [10]. It can reduce young people’s capacity to undertake healthy behaviours [11]. These impacts may persist into adulthood and increase risk of chronic conditions.

Addressing weight stigma among adolescents has been identified as critical for the physical and mental health of future generations [12]. However, evidence on preventing and reducing weight stigma is underdeveloped and methodological inconsistency impedes comparisons on intervention effectiveness [13]. Moreover, available evidence relates almost entirely to HICs, while LMIC research has been sparse, despite escalating OW and obesity prevalence. We undertook formative research to provide insight into the extent and experience of weight stigma among adolescents in three LMICs and identified young people’s recommendations for action.

## METHODS

### Setting

The study was conducted in South Africa, Indonesia, and Brazil. Country selection was pragmatic, based on the availability of market research databases from which to recruit young people, national COVID-19 situations, and countries where OW and obesity prevention is a UNICEF priority. Estimated OW and obesity prevalence among children and adolescents aged under 20 years ranged from 35.5% in Brazil, 33.7% in South Africa, and 24.7% in Indonesia in 2021 [14].

### Sampling

We used a large national market research agency database to identify households including adolescents and connected with households via a simple randomization method. We undertook a screening telephone call with the adult database contact to confirm study eligibility (the presence of adolescents aged 15-19 years, telephone access) and to record demographics, including the adolescent’s self-assessed weight (HW, OW, underweight (UW)). Households that fulfilled the criteria, irrespective of self-reported weight, were included in the sampling framework and selected at random for survey or interview invitation. Of all the respondents who agreed to participate, the percentage of young people that subsequently dropped out was 22.3% in Brazil, 26.9% in Indonesia, and 7.9% in South Africa. Parental permission was sought; parents and adolescents received verbal information and were asked for consent (>18 years) or assent (<18 years). Sample distribution was equal in sex, urban/rural, age bands, and between countries.

### Data collection

#### Preliminary data

To guide the survey design, we undertook a rapid literature review to identify major themes and issues in existing research and found that LMIC weight stigma research is sparse. Some studies related to weight perceptions exist for some middle-income countries, yet this research does not address weight stigma, and published prevalence and experience-related research is lacking. Based on the findings of our rapid review and on the advice of a stigma expert, we conducted exploratory in-depth semi-structured interviews (n = 18; six per country) to assess whether the issue resonated with LMIC adolescents and identify similarities and differences compared to prior research, which informed survey design. The research team undertook the interviews via the Zoom online platform in the interviewees’ preferred language (Zulu, Bahasa Indonesia, and Portuguese), which were recorded, transcribed, and translated by the research team. We used the qualitative data primarily to guide the quantitative survey design (described below). In presenting the results, we used qualitative interview quotations to illustrate themes and give insight into how adolescents expressed their experiences or views.

#### Quantitative survey

A telephone survey was undertaken with a cohort of adolescents (n = 1200; 400 per country). Computer-assisted telephonic interviewing (CATI) technology was used, whereby the interviewer recorded responses immediately into a database. Interviewers used a structured questionnaire with primarily closed and a small number of open-ended questions. The survey included questions about young people’s weight stigma related experiences, impacts, and recommendations, alongside selected questions from two validated scales that have been used to measure specific aspects of weight stigma. Questions on weight stigma internalization were taken from an adapted version of the Weight Bias Internalization Scale – whether the interviewee was happy with their weight or not; and the extent to which their weight perception influenced their self-esteem [12]. Questions from the Attitudes Towards Obese Persons scale were included – whether the interviewee perceived that people affected by OW were as successful, attractive, and had as much ‘self-control’ as others; and how comfortable people feel when associating with people affected by OW [13]. The survey was translated (Portuguese in Brazil; Bahasa Indonesia in Indonesia; and Zulu, Xhosa, and Afrikaans for South Africa) and interviews were conducted with consenting participants. Interviewers received training on using the survey instrument.

#### Data analysis and management

Survey data were collected via Decipher, an electronic and data management platform, which was password-protected and securely stored on the RTI Enhanced Security Network. Both qualitative and quantitative data were deidentified before analysis to protect participant privacy and were accessed only by research team members. Data will be archived for three years following project conclusion, after which all electronic data will be destroyed. The study was approved the RTI International Institutional Research Board as a social and behavioural study (STUDY00021600) on July 7, 2021.

Thematic analysis of the preliminary qualitative data was conducted manually. Data were coded in Microsoft Excel and findings were searched and cross-referenced to identify themes, areas of consensus, commonalities, and differences between respondents, and within and between countries. A coding frame was agreed upon by the research team and coding was simultaneously undertaken by two researchers. SPSS-23 was used to analyse the quantitative survey data. Descriptive analysis was undertaken for sample demographics and characteristics and for all outcome indicators, including the percentage distribution of categorical variables. Outcome data has been presented by respondent sub-group (sex, weight category, and area of residence).

## RESULTS

522 males and 678 female adolescents participated in the survey (**Table 1**). More females ( ~ 60%) responded in South Africa and Brazil compared to Indonesia, where responses were evenly spread. The average age was similar across countries. Urban and rural dwellers were well represented, with somewhat greater participation from urban dwellers. Participants were asked to self-classify their weight as “healthy weight”, “overweight”, or “underweight” – a method shown to be a reliable proxy for objective measurement [12]. More than one in three South African respondents identified themselves as affected by OW, one in four in Brazil, and approximately one in two in Indonesia.

**Table 1:** Participants’ characteristics*

	South Africa	Brazil	Indonesia	Total
**Sex**				
Male	157 (39.3)	158 (39.5)	207 (51.8)	522 (43.5)
Female	243 (60.8)	242 (60.5)	193 (48.3)	678 (56.5)
**Age (years)**				
15	85 (21.3)	64 (16.0)	79 (19.8)	228 (19.0)
16	86 (21.5)	78 (19.5)	84 (21.0)	248 (20.7)
17	88 (22.0)	83 (20.8)	83 (20.8)	254 (21.2)
18	74 (18.5)	104 (26.0)	72 (18.0)	250 (20.8)
19	67 (16.8)	71 (17.8)	82 (20.5)	220 (18.3)
Mean age ± SD	16.9 ± 1.38	17.1 ± 1.34	16.9 ± 1.41	17 ± 1.38
**Area**				
Rural	182 (45.5)	152 (38.0)	185 (46.3)	519 (43.3)
Urban	218 (54.5)	248 (62.0)	215 (53.8)	681 (56.8)
**Weight of the respondent as categorized by them**				
HW	187 (46 · .)	259 (64.8)	103 (25.8)	549 (45.8)
OW	156 (39.0)	97 (24.3)	182 (45.5)	435 (36.3)
UW	57 (14.3)	54 (11.0)	115 (28.8)	216 (18.0)
**Total sample**	400	400	400	1200

HW – healthy weight, OW – overweight, UW – underweight

*Unless otherwise stated, the absolute numbers present the number of respondents for each country by category and the figures in parentheses represent the corresponding percent.

### Experience of weight stigma

A large proportion of participants in South Africa and Brazil who had assessed themselves as being affected by OW received negative weight-related comments. Females (South Africa = 73.3%; Brazil = 92.6%) were subjected to more comments than males (South Africa = 64.7%; Brazil = 75.9%), and adolescents residing in urban areas reported the most comments. Sources varied by sex and geography. In South Africa, males reported schoolmates as the most frequent source of negative weight-related comments; for females, it was family members. In urban areas, negative comments were more likely to come from a family member, while in rural areas, schoolmates were more frequently cited. In parallel with South Africa, Brazilian males identified schoolmates as the most common source, whereas females highlighted family members. The urban-rural pattern in South Africa and Brazil was similar.

The situation was different in Indonesia. Like South Africa and Brazil, more than half the males affected by OW (53.8%) had received negative weight-related comments, but the proportion was lower among females (46.2%). Another difference was that more Indonesian males said they were not bothered by weight-related comments compared to the other countries.

Notably, even adolescents with HW and those affected by UW could receive negative weight-related comments. This was highest in Brazil among males (HW = 63.1%; UW – 88 · 9%) and females (HW = 83.1%; UW = 84.6%). In South Africa and Indonesia, the proportions of adolescents of HW or affected by UW that received negative comments were lower, with females receiving more comments (South Africa: HW = 43.7%; UW = 45.7%; Indonesia: HW = 17.6%; UW = 56.3%) than males (South Africa: HW = 22.6%; UW = 40.9%; Indonesia: HW = 13.5%; UW = 37.3%).

### Internalization of weight stigma

**Figure 1:**
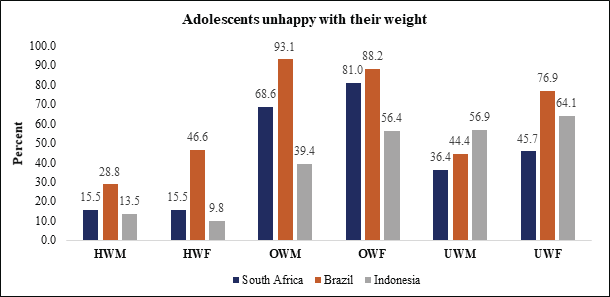
Dissatisfaction among adolescents pertaining to their weight.

In South Africa and Brazil, adolescents affected by OW were unhappy with their weight. This was consistent across rural and urban areas. In Indonesia, males were relatively happier with their weight (60.6%), while more than half the females (56.4%) wanted to change their weight. Among those affected by UW, males in Indonesia (56.9%) and females in Brazil (76.9%) were unhappiest. Among those of HW, unhappiness was low across all three countries. Overall, weight-related dissatisfaction was substantially higher among adolescents affected by OW (**Figure 1**). However, notable dissatisfaction was found among adolescents affected by UW, and weight unhappiness in Brazil also extended to adolescents of HW.

Adolescents affected by OW were asked about the influence of their weight on their self-perception. The largest proportion of South African participants indicated a negative impact (males = 41.2%; females = 46.7%). While most females in Brazil said it contributed to negative self-perception (52.9%), only one-fifth of males reported this (20.7%). Indication of such weight stigma internalization was considerably less in Indonesia (males = 14.4%; females = 17 · 9%).

“That comment, ‘Wow! you are different’, ‘you are a little bit fat, or fatter’, these comments never offended me, but the comments. ‘Soon you will no longer fit into clothes, you will have to make clothes’, ‘You will no longer pass through the entrance of a bus’. These comments, sometimes I ended up not answering, but I didn't like it” (Brazil, female, 18 years, affected by OW).

Among those affected by UW, negative self-perception was highest among females in Brazil (53.8%), while in South Africa and Indonesia, it was less than one-fifth of males and females. Adolescents of HW had little or no negative weight-related perception, except in Brazil, where one-quarter of females felt this (24.3%).

Adolescents across all weight categories did not consider their weight status a barrier to success and considered themselves as attractive as people of other weight statuses. Similar patterns were observed in urban and rural areas and between males and females. Nonetheless, other measures used to identify internalized weight stigma suggested that negative weight-related perceptions were more common, particularly in relation to OW.

Thus, female adolescents believed people affected by OW had less self-control (South Africa = 44.8%; Brazil = 26.5%; Indonesia = 41.0%) compared to males. Among those with HW, females in South Africa and Indonesia shared that belief (South Africa = 46.6%; Indonesia = 31.4%) as did Brazilian males (Brazil = 36%). Males affected by UW across all three countries (South Africa = 50.0%; Brazil = 22.2%; Indonesia = 47.1%) consistently believed that people affected by OW had less self-control, compared to females (South Africa = 28.6%; Brazil = 11.5%; Indonesia = 31.3%).

Most adolescents affected by OW in South Africa and Brazil believed that other people feel uncomfortable associating themselves with someone affected by OW (South Africa: males = 39.2%; females = 46.9%; Brazil: males = 51.7%; females = 60.3%). This proportion was far less in Indonesia (males = 12.5%; females = 10.3%). Similar beliefs were also found among adolescents with HW and those affected by UW in South Africa and Brazil, but not in Indonesia.

### Impacts of weight stigma on young people

When adolescents received negative comments, it made them unhappy, irrespective of their weight status. One in five (20.2%) of the total sample of 1200 adolescents reported adverse impacts from such comments, relating to their mental health and weight perception and eating behaviour. A small minority reported that comments gave them a positive impetus for healthy behaviours.

“There would always be that one person who would bring you down or would make a joke about it. It would affect you in so many ways. I have very low self-esteem and I have a lot of insecurities about myself. And like if someone would just say, like one small silly thing, I would actually, I would really feel bad.” (South Africa, female, 16 years, OW).

Most of the negative impacts reported by adolescents related to mental health (71.8%), describing (for example) anxiety, depression, insecurity, self-doubt, and low self-confidence. The second greatest impact area was negative weight perceptions and eating behaviour (23.1%), including reduced appetite, anorexia, binge eating, and feeling ashamed of their weight. Very few (5.1%) responses suggested that comments had encouraged young people towards positive health behaviours, such as undertaking regular exercise.

Weight clearly was a very sensitive topic. While more adolescents affected by OW experienced this, the impacts of weight stigma could be substantial and intense among young people across all weight categories.

“It makes me less confident. The comments and things are breaking you down and you're feeling not confident, and you don't even feel like talking to people because, as it is everybody's judging you.” (South Africa, male, 17 years, affected by OW).

### Young people’s recommendations

Young people across all countries and weight categories emphasized the importance of addressing weight-related bullying in schools by promoting a more inclusive model of health and body image. They believed this would promote respect and understanding between people of different shapes and sizes and motivate and encourage everyone to optimize their health without blame or exclusion.

“It is actually okay for us to love our weight, whatever the number is. And we shouldn’t judge others based on their weight whatever the number is.” (Indonesia, Female, 17 years, affected by underweight).

Participants identified the need for support to achieve and maintain a HW. Frequent recommendations by young people of all weight categories and all countries included: the provision of non-judgmental, easily accessible advice, information and support for young people struggling with their weight, and alleviating the counter-productive pressure that was perceived to feature strongly in existing advertising campaigns and media for example for junk food. Digital communication and social media were the most frequently recommended dissemination channels for information, advice, and supportive resources in all countries (South Africa: TikTok and Facebook; Brazil and Indonesia: Instagram and YouTube) and were equally popular in urban and rural areas.

“The content has to be online – on social media. Information about how to live a healthy lifestyle, healthy recipes – how people can make them, and exercises that can be practiced at home or at school can be shown.” (South Africa, female, 19 years, HW).

Participants advocated for more supportive environments where it is easier to make healthier choices. They highlighted the perceived greater cost of healthy products or undertaking certain activities, and bombardment with fast food marketing and easy availability. Adolescents recognized the dissonance between this and the dominant promotion of thinness as the preferred body type.

“Sometimes it also depends on money. Because to go to the gym you have to spend $60 a month, to buy things to lose weight, which is a little more expensive, you have to spend $20 on one thing, and then another $50 on something else… If you buy some fruit...” (Brazil, male, 16 years, affected by OW).

Young people said COVID-19 had impacted their weight and their ability to manage this. For most, the impact was negative: reduced physical activity opportunities when leisure venues closed and during lockdowns, and home-schooling without physical activity or commuting. Young people affected by OW reported that loss of routine, being cut off from friends and schoolmates, and boredom adversely affected their diet, with increased snacking and weight gain, or for some, unhealthy weight loss.

“At the beginning of the pandemic we didn't have classes for a few months and my routine became very... I spent the whole night on my cell phone, I would wake up late, I wanted to eat snacks, I only watched movies. I gained a lot of weight and then, from September I had a lot of anxiety crisis and became bad, very thin, with a bad diet.” (Brazil, female, 15 years, HW).

## DISCUSSION

This study, with a diverse sample of adolescents aged 15-19 years in three LMICs, found that many had experienced weight stigma – particularly adolescents affected by OW – and that the impacts were overwhelmingly negative. Weight stigma undermined the affected adolescents’ mental health, fostered negative weight perceptions, and could engender disordered eating behaviour. This echoes the broad pattern of previous weight stigma research among adolescents and the wider population in HICs [15]. In the context of the rapidly escalating rates of OW and obesity among adolescents in LMICs [16] the leading implication of this study’s findings is that the issue of weight stigma and its adverse impacts on young people’s mental and physical health requires policy attention and practical action.

This study also identified that weight stigma could also adversely impact young people affected by UW. This theme is not a major feature of existing research, which has largely been conducted in HICs, where the proportions of adolescents affected by UW are considerably less than in LMICs, which experience the “double-burden of malnutrition” [17]. HIC research primarily indicates weight stigma of adolescents affected by OW as a risk factor for eating disorders [18], but studies of adolescents affected by eating disorders suggest that UW can also be perceived as stigmatizing [19].

We found that large proportions of adolescents in each country (males and females) reported weight dissatisfaction, vocalizing an internalized belief that their body did not look or feel the way that it was expected to, both to others and themselves. This negative self-perception was most common among adolescents affected by OW, with more than half of all respondents (male and female) across all three countries expressing unhappiness with their weight. Prior global research suggests a linear relationship between perceptions of OW and population prevalence [20], and this study’s findings illustrate that the increasing rates of OW in LMICs are translating into more widespread negative weight perceptions. Previous research indicates that weight dissatisfaction can adversely impact mental and physical well-being, manifesting in binge eating, weight gain, and social isolation, and can reduce the capacity to undertake health-promoting behaviours, such as physical activity [21]. Some young people in this study reported that weight stigma, alongside their own negative internalized perceptions, had contributed to unhealthy eating patterns and undermined their ability to follow a healthy diet.

Regarding gender, research in HICs indicates that both young women and young men may experience weight stigma, but prevalence is greater among females [15]. This study concurs in finding that more females experienced weight stigma, however, differs in suggesting that weight stigma may be a more common experience among young men than previously indicated – at least in LMICs. Moreover, the reported level of weight-related unhappiness was substantial among males of various weight statuses in different countries, and more Brazilian males affected by OW were unhappy with their weight compared to any sub-group.

Investment in programs to promote mental well-being and prevent mental illness among adolescents in LMICs has been assessed as insufficient, and poor access to care for common and increasingly prevalent mental disorders, such as depression and anxiety, remains widespread [22] Additionally, recently published research suggests the COVID-19 pandemic adversely impacted adolescent mental health worldwide. Studies also suggest that the pandemic undermined some people’s ability to manage their weight via a healthy diet and physical activity [23], and may have increased weight stigmatization [24]. These interlinked challenges were reported by adolescents in this study, reinforcing the main finding that weight stigma is undermining the mental well-being of many adolescents in different countries. This bolsters the case for governments to give adolescent mental health higher priority and to address the obesity-mental health nexus.

Adolescents’ strongest recommendation was for robust action to prevent and reduce weight stigma. Like in HIC studies, school was a major locus of weight stigma. However, LMIC adolescents in this study indicated that their peers were the sole enactors at school, whereas HIC studies also suggested that teachers sometimes enacted weight stigma [25]. Some examples of generalized anti-bullying initiatives exist in schools, and this is encouraged under the WHO Health Promoting Schools initiative and by UNICEF (United Nations Children’s Fund). Programs to promote healthier school food environments are another existing policy strand in many countries. Incorporating action against weight stigma within existing food, mental health, and anti-bullying initiatives, and developing new programs where none exist, including issues in curricula, and teacher training offer tangible avenues for action.

### Limitations

The study included three LMICs, with the participants representing substantial cultural, regional, and social diversity. However, future findings may potentially differ in other countries. It is also possible that participants differed in certain respects from non-participants, or that their experience of weight stigma differed from adolescents more generally. Nonetheless, the study purposively included 1200 adolescents of wide-ranging characteristics, which lends confidence that the findings present a reliable account of adolescent experiences within this age cohort in the three countries.

## CONCLUSIONS

As LMIC stakeholders increasingly become aware and seek to respond to increasing OW and obesity prevalence and its associated burden of chronic disease, our findings suggest that addressing weight stigma will be vital. Researchers and health organizations in HICs have advocated for national obesity prevention and reduction, suggesting strategies and interventions should be designed to avoid weight stigmatization. Our findings suggest this issue is equally pertinent to LMICs; besides ensuring weight-related interventions are non-stigmatizing, policymakers and practitioners across sectors should proactively counter weight stigma and place greater emphasis on mental well-being. Young people have suggestions about how this can be done and should be involved in intervention design. Recognizing weight stigma as a public health concern and taking measures to address this will benefit countries’ efforts to prevent and reduce NCDs among young people now and in the future.
